# Supplementary material for: Benchmarking metabolic RNA labeling techniques for high-throughput single-cell RNA sequencing
Source: Nat Commun. 2025 Jul 1;16:5952. doi: 10.1038/s41467-025-61375-z (PMC12215390; doi:10.1038/s41467-025-61375-z)
Supplement: Supplementary file 1 — Supplementary Information [file 41467_2025_61375_MOESM1_ESM.pdf]

Supplementary Fig. 1

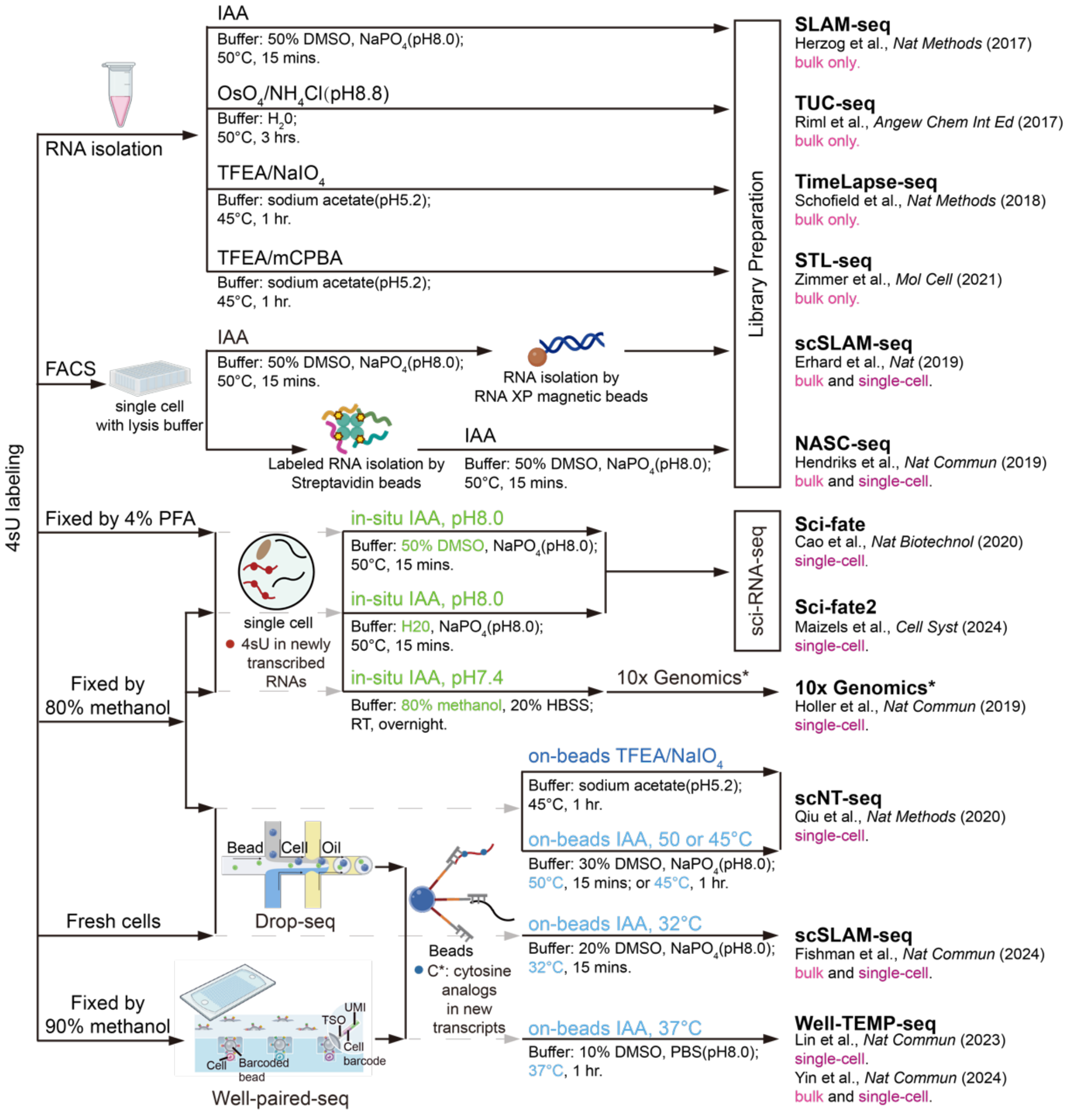

*Except for 10x Genomics, which is labeled with 4sUTP, the other methods are all labeled with 4sU.

FACS: Fluorescence Activated Cell Sorting; PFA: paraformaldehyde;
DMSO: dimethylsulfoxide; $NaPO_4$: sodium phosphate;
IAA: iodoacetamide; TFEA: 2,2,2-trifluoroethylamine; $NaIO_4$: sodium periodate;
mCPBA: meta-chloroperoxybenzoic acid; OsO_4: osmium tetroxide; NH_4Cl: ammonium chloride;
4sUTP: 4-thiouridine-triphosphate; 4sU: 4-Thiouridine.

Supplementary Fig. 1 | Summary of the chemical conversion methods.

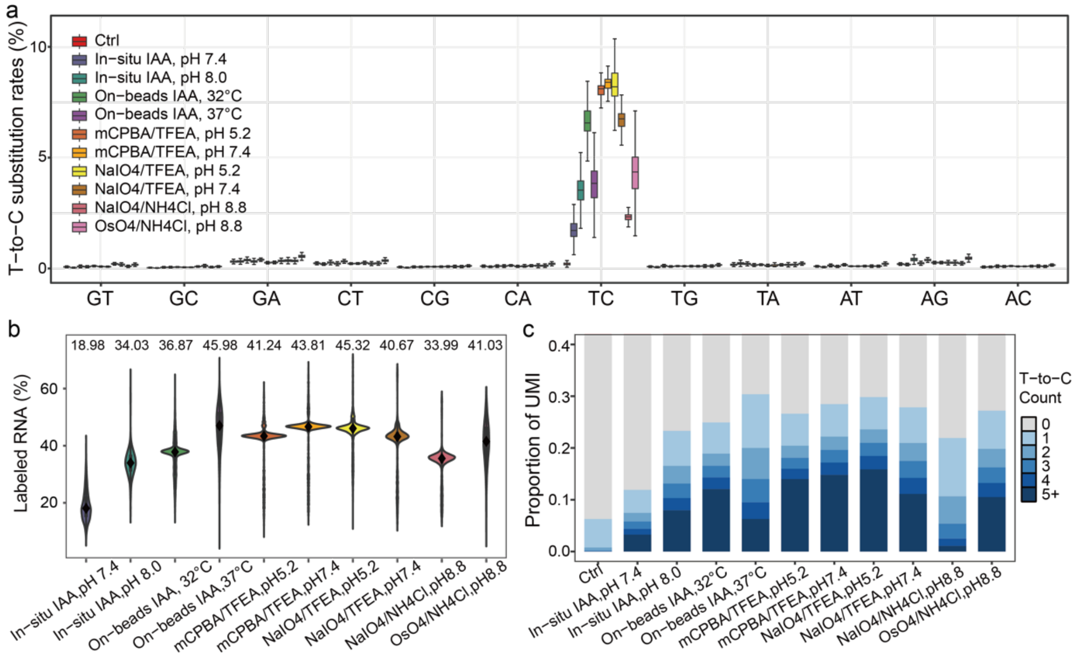

d

Methods		cDNA size (bp)
Ctrl	Without chemical conversion	1,103
SLAM-based	In-situ IAA, pH 7.4	985
	In-situ IAA, pH 8.0	1,039
	On-beads IAA, 32 °C	1,031
	On-beads IAA, 37 °C	1,072
TimeLapse-based	mCBPA/TFEA, pH 5.2	1,091
	mCPBA/TFEA, pH 7.4	1,025
	NaIO4/TFEA, pH 5.2	837
	NaIO4/TFEA, pH 7.4	922
	NaIO4/NH4Cl, pH 8.8	702
TUC-based	OsO4/NH4Cl, pH 8.8	965

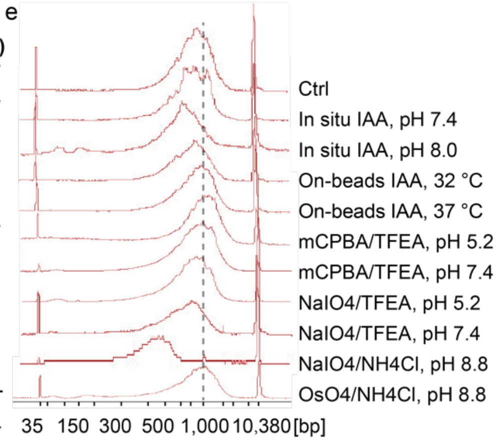

Supplementary Fig. 2 | Comparison of ten chemical conversion methods.

a. Box plot showing nucleotide substitution rates across ten chemical conversion methods in 4sU-labeled ZF4 cells. "Ctrl" represents the control group without chemical treatment. Different colored boxes represent different treatment methods, with the box edges indicating the 25th and 75th percentiles. Source data are provided as a Source Data file.

b. Violin plots showing the distribution of labeled RNA fractions across the ten chemical conversion methods. Color indicates treatment methods. The central dot indicates the median, and the box edges indicate the 25th and 75th percentiles. The mean of each group is displayed at the top. Source data are provided as a Source Data file.

c. Proportion of UMIs containing T-to-C substitutions under ten chemical conversion methods. The color gradient indicates the number of T-to-C substitutions per read, with darker shades representing a higher number of substitutions within the UMI. "Ctrl" represents the control group without chemical treatment. Source data are provided as a Source Data file.

d. Summary of cDNA library sizes for the control sample without chemical conversion and across the ten chemical conversion methods.

e. Peak size distribution of cDNA libraries measured using the 2100 Bioanalyzer.

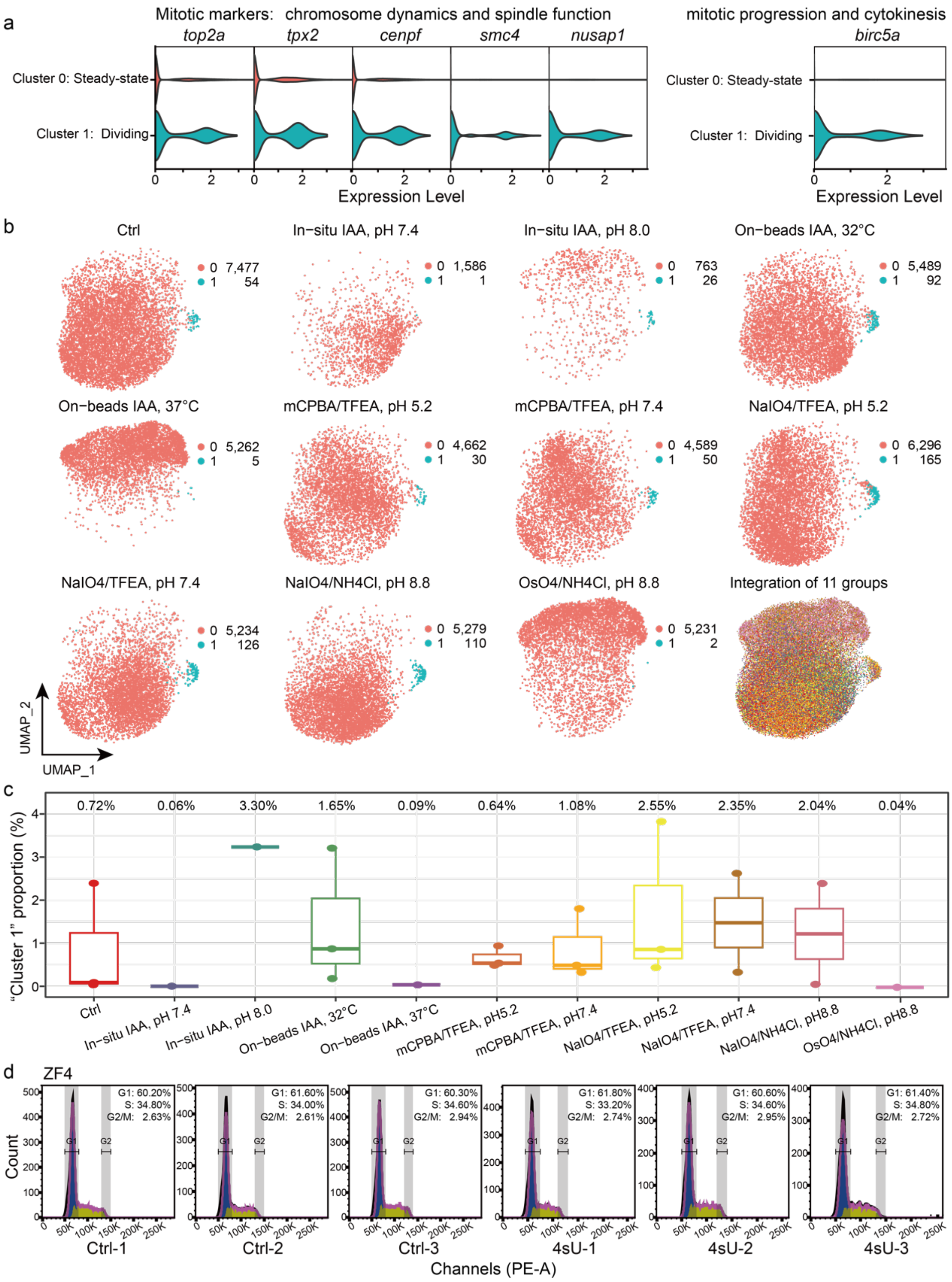
a
Mitotic markers: chromosome dynamics and spindle function
mitotic progression and cytokinesis
top2a
tpx2
cenpf
smc4
nusap1
birc5a
Cluster 0: Steady-state
Cluster 1: Dividing
Expression Level
b
Ctrl
0 7,477
1 54
In−situ IAA, pH 7.4
0 1,586
1 1
In−situ IAA, pH 8.0
0 763
1 26
On−beads IAA, 32°C
0 5,489
1 92
On−beads IAA, 37°C
0 5,262
1 5
mCPBA/TFEA, pH 5.2
0 4,662
1 30
mCPBA/TFEA, pH 7.4
0 4,589
1 50
NaIO4/TFEA, pH 5.2
0 6,296
1 165
NaIO4/TFEA, pH 7.4
0 5,234
1 126
NaIO4/NH4Cl, pH 8.8
0 5,279
1 110
OsO4/NH4Cl, pH 8.8
0 5,231
1 2
Integration of 11 groups
UMAP_2
UMAP_1
c
“Cluster 1” proportion (%)
0.72%
0.06%
3.30%
1.65%
0.09%
0.64%
1.08%
2.55%
2.35%
2.04%
0.04%
Ctrl
In−situ IAA, pH 7.4
In−situ IAA, pH 8.0
On−beads IAA, 32°C
On−beads IAA, 37°C
mCPBA/TFEA, pH5.2
mCPBA/TFEA, pH7.4
NaIO4/TFEA, pH5.2
NaIO4/TFEA, pH7.4
NaIO4/NH4Cl, pH8.8
OsO4/NH4Cl, pH8.8
d
ZF4
Count
G1: 60.20% S: 34.80% G2/M: 2.63%
G1: 61.60% S: 34.00% G2/M: 2.61%
G1: 60.30% S: 34.60% G2/M: 2.94%
G1: 61.80% S: 33.20% G2/M: 2.74%
G1: 60.60% S: 34.60% G2/M: 2.95%
G1: 61.40% S: 34.80% G2/M: 2.72%
Ctrl-1
Ctrl-2
Ctrl-3
4sU-1
4sU-2
4sU-3
Channels (PE-A)

Supplementary Fig. 3 | The proportion of different cell states.

a. Violin plots showing the expression of well-known mitotic cell cycle genes in different cluster. “0” represent cluster “steady-state”; “1” represent cluster “dividing”. The central dot indicates the median, and the box edges indicate the 25th and 75th percentiles. *top2a*, *tpx2*, *cenpf*, *smc4*, and *nusap1* are mitotic markers involved in chromosome dynamics and spindle function; *birc5a* (Survivin) is involved in both mitotic progression and cytokinesis.

b. Uniform Manifold Approximation and Projection (UMAP) visualization showing datasets respectively from control and the ten chemical conversion methods. "Ctrl" represents the control group without chemical treatment. Cells are colored by cell type (top left 11 groups) or by the corresponding chemical conversion method (lower right corner).

c. Box plot showing dividing cluster proportion across control and the ten chemical conversion methods in 4sU-labeled ZF4 cells. "Ctrl" represents the control group without chemical treatment. Different colored boxes represent different treatment methods, with the box edges indicating the 25th and 75th percentiles. Source data are provided as a Source Data file.

d. Flow cytometry analysis of cell cycle distribution in ZF4 cells. Cells were either untreated (left three panels) or treated with 4sU (right three panels) and stained with PI to assess DNA content. Histograms display the distribution of cells across G1, S, and G2/M phases. "Ctrl" represents the untreated control group, while "4sU" denotes the labeled group with 4sU treatment.

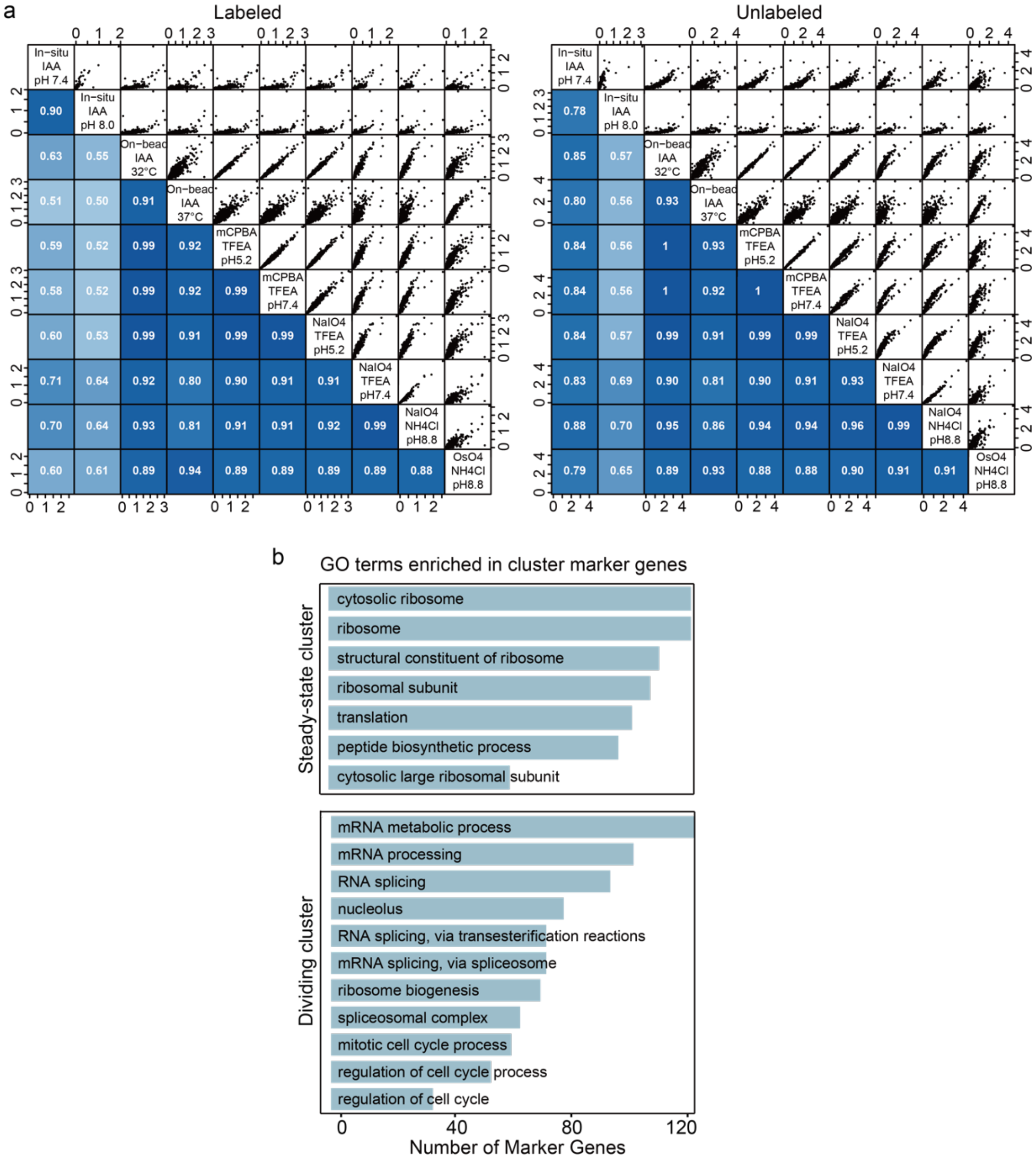

a
Labeled
Unlabeled
In−situ IAA pH 7.4
In−situ IAA pH 8.0
On−bead IAA 32°C
On−bead IAA 37°C
mCPBA TFEA pH5.2
mCPBA TFEA pH7.4
NaIO4 TFEA pH5.2
NaIO4 TFEA pH7.4
NaIO4 NH4Cl pH8.8
OsO4 NH4Cl pH8.8
b
GO terms enriched in cluster marker genes
Steady-state cluster
cytosolic ribosome
ribosome
structural constituent of ribosome
ribosomal subunit
translation
peptide biosynthetic process
cytosolic large ribosomal subunit
Dividing cluster
mRNA metabolic process
mRNA processing
RNA splicing
nucleolus
RNA splicing, via transesterification reactions
mRNA splicing, via spliceosome
ribosome biogenesis
spliceosomal complex
mitotic cell cycle process
regulation of cell cycle process
regulation of cell cycle
0
40
80
120
Number of Marker Genes

Supplementary Fig. 4 | mRNA control strategy during the cell cycle.

a. Scatterplots (upper right) illustrating Pearson's correlation between the abundances of labeled and unlabeled RNA across the ten chemical conversion methods. The heatmap (lower left) indicates the transcriptional expression correlation across different methods, with numerical values representing the Pearson correlation coefficient. The darker the color, the higher the correlation. RNA expression levels are presented as the natural logarithm of (TP10K + 1).

b. Gene Ontology term enrichment analysis for the top 100 marker genes identified in both steady-state and dividing cell clusters. Significance was assessed using a one-sided hypergeometric test, with bar lengths representing the number of enriched genes.

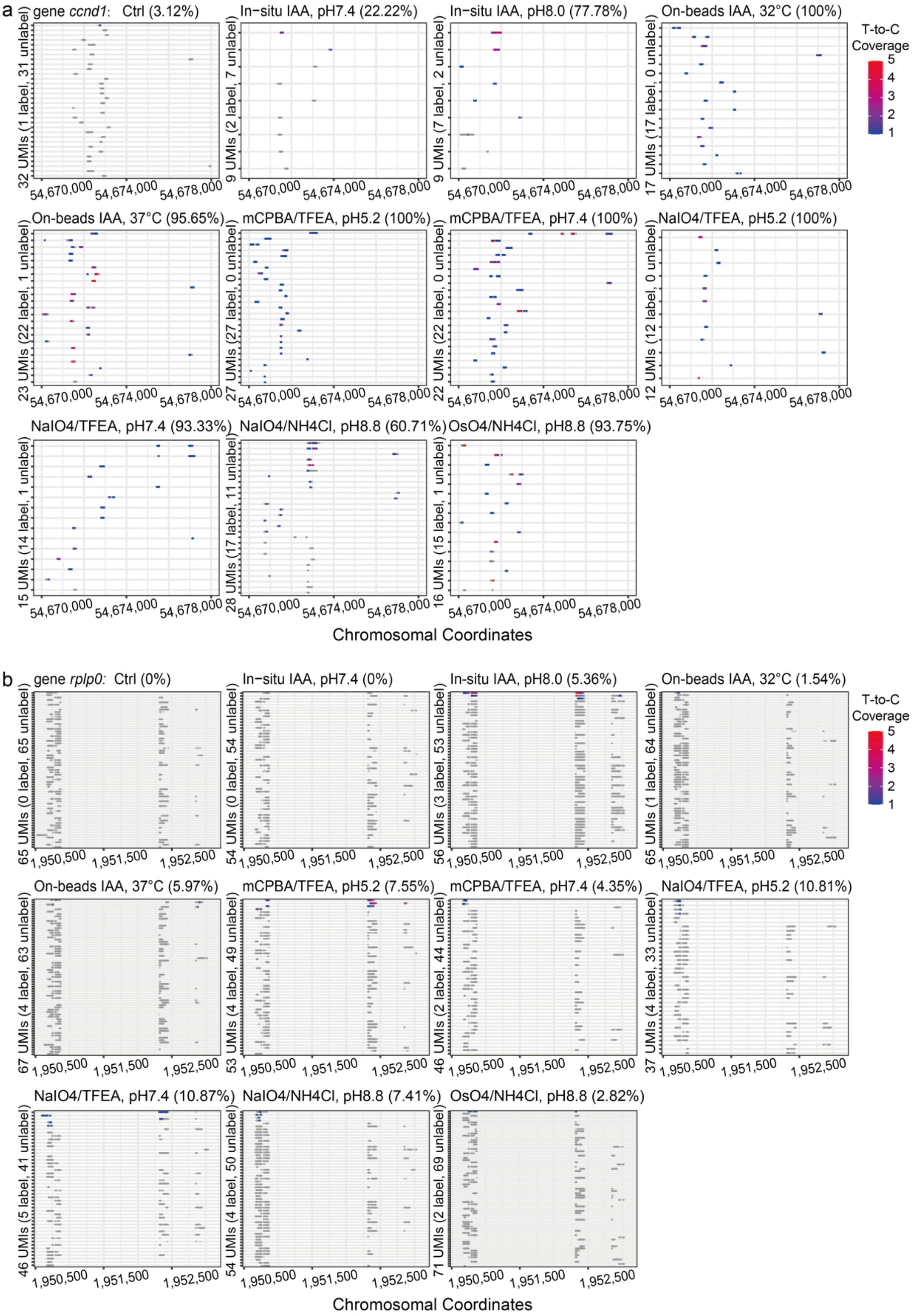
a
gene ccnd1: Ctrl (3.12%)
In−situ IAA, pH7.4 (22.22%)
In−situ IAA, pH8.0 (77.78%)
On-beads IAA, 32°C (100%)
T-to-C Coverage
5
4
3
2
1
32 UMIs (1 label, 31 unlabel)
9 UMIs (2 label, 7 unlabel)
9 UMIs (7 label, 2 unlabel)
17 UMIs (17 label, 0 unlabel)
On-beads IAA, 37°C (95.65%)
mCPBA/TFEA, pH5.2 (100%)
mCPBA/TFEA, pH7.4 (100%)
NaIO4/TFEA, pH5.2 (100%)
23 UMIs (22 label, 1 unlabel)
27 UMIs (27 label, 0 unlabel)
22 UMIs (22 label, 0 unlabel)
12 UMIs (12 label, 0 unlabel)
NaIO4/TFEA, pH7.4 (93.33%)
NaIO4/NH4Cl, pH8.8 (60.71%)
OsO4/NH4Cl, pH8.8 (93.75%)
15 UMIs (14 label, 1 unlabel)
28 UMIs (17 label, 11 unlabel)
16 UMIs (15 label, 1 unlabel)
54,670,000
54,674,000
54,678,000
Chromosomal Coordinates
b
gene rplp0: Ctrl (0%)
In−situ IAA, pH7.4 (0%)
In−situ IAA, pH8.0 (5.36%)
On-beads IAA, 32°C (1.54%)
T-to-C Coverage
5
4
3
2
1
65 UMIs (0 label, 65 unlabel)
54 UMIs (0 label, 54 unlabel)
56 UMIs (3 label, 53 unlabel)
65 UMIs (1 label, 64 unlabel)
On-beads IAA, 37°C (5.97%)
mCPBA/TFEA, pH5.2 (7.55%)
mCPBA/TFEA, pH7.4 (4.35%)
NaIO4/TFEA, pH5.2 (10.81%)
67 UMIs (4 label, 63 unlabel)
53 UMIs (4 label, 49 unlabel)
46 UMIs (2 label, 44 unlabel)
37 UMIs (4 label, 33 unlabel)
NaIO4/TFEA, pH7.4 (10.87%)
NaIO4/NH4Cl, pH8.8 (7.41%)
OsO4/NH4Cl, pH8.8 (2.82%)
46 UMIs (5 label, 41 unlabel)
54 UMIs (4 label, 50 unlabel)
71 UMIs (2 label, 69 unlabel)
1,950,500
1,951,500
1,952,500
Chromosomal Coordinates

Supplementary Fig. 5 | Identification of newly transcribed cell cycle genes in ZF4 cells.
a-b. Visualization of unique transcripts (with unique UMIs) for the cell-cycle gene *ccnd1* (a) and the housekeeping gene *rplp0* (b) from individual ZF4 cells in the control group and across the ten chemical conversion methods. Grey circles represent uridines without T-to-C substitution, while crosses ("X"s) indicate uridines with T-to-C substitutions in at least one read. The color scale represents the read coverage for each T-to-C substitution event.

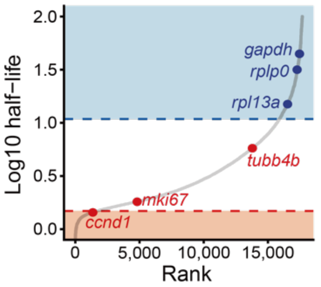

	Description	p.adjust
Bottom10% (Stable)	Oxidative phosphorylation	6.59E-18
	Respirasome	1.19E-12
	Cellular respiration	2.19E-10
	Oxidoreductase complex	3.79E-07
Top 10% (Unstable)	DNA-binding transcription activator activity	1.91E-04
	Cell cycle	5.95E-04
	Transcription regulator complex	9.11E-04
	Positive regulation of RNA biosynthetic process	1.25E-03

Supplementary Fig. 6. The half-lives of detected genes in steady-state cluster.

Dot plot showing the ranking of the half-lives of detected genes in steady-state cluster, with cell cycle and housekeeping genes highlighted (left panel). The table displays the significantly enriched Gene Ontology terms for the top 10% of genes with the shortest half-lives and the bottom 10% with the longest half-lives, along with their adjusted P-values (right panel). Significance was determined using a hypergeometric test with FDR adjustment.

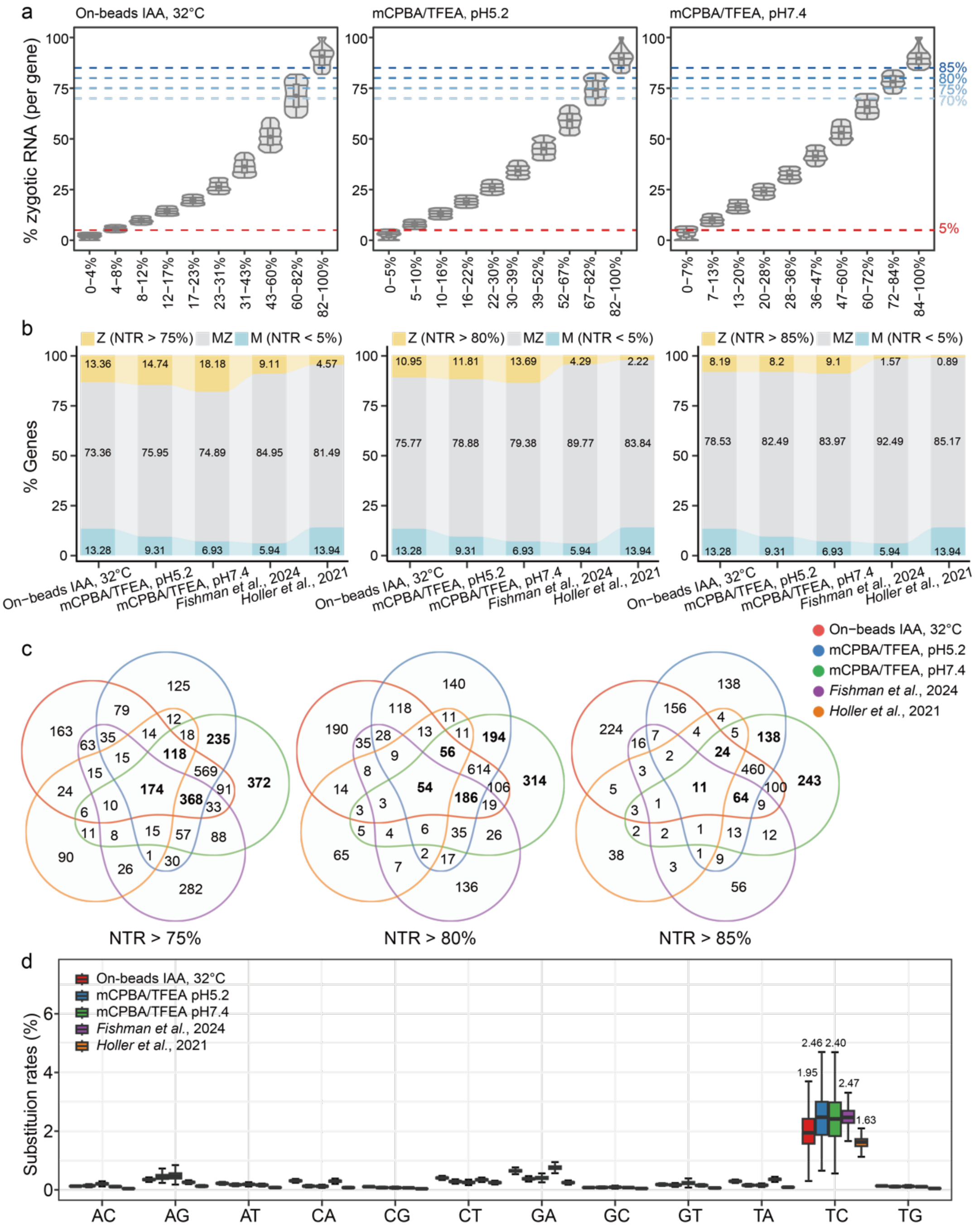
a
On-beads IAA, 32°C
mCPBA/TFEA, pH5.2
mCPBA/TFEA, pH7.4
% zygotic RNA (per gene)
85%
80%
75%
70%
5%
0–4% 4–8% 8–12% 12–17% 17–23% 23–31% 31–43% 43–60% 60–82% 82–100%
0–5% 5–10% 10–16% 16–22% 22–30% 30–39% 39–52% 52–67% 67–82% 82–100%
0–7% 7–13% 13–20% 20–28% 28–36% 36–47% 47–60% 60–72% 72–84% 84–100%
b
Z (NTR > 75%) MZ M (NTR < 5%)
Z (NTR > 80%) MZ M (NTR < 5%)
Z (NTR > 85%) MZ M (NTR < 5%)
% Genes
13.36 14.74 18.18 9.11 4.57
73.36 75.95 74.89 84.95 81.49
10.95 11.81 13.69 4.29 2.22
75.77 78.88 79.38 89.77 83.84
8.19 8.2 9.1 1.57 0.89
78.53 82.49 83.97 92.49 85.17
13.28 9.31 6.93 5.94 13.94
On−beads IAA, 32°C
mCPBA/TFEA, pH5.2
mCPBA/TFEA, pH7.4
Fishman et al., 2024
Holler et al., 2021
c
On−beads IAA, 32°C
mCPBA/TFEA, pH5.2
mCPBA/TFEA, pH7.4
Fishman et al., 2024
Holler et al., 2021
NTR > 75%
NTR > 80%
NTR > 85%
d
On-beads IAA, 32°C
mCPBA/TFEA pH5.2
mCPBA/TFEA pH7.4
Fishman et al., 2024
Holler et al., 2021
Substitution rates (%)
1.95 2.46 2.40 2.47 1.63
AC AG AT CA CG CT GA GC GT TA TC TG

Supplementary Fig. 7 | Application of metabolic labeling scRNA-seq to identify zygotically activated transcripts in zebrafish.

a. Violin plots showing the percentage of zygotic mRNA per gene across all expressing cells (y-axis). Genes were grouped into 10 bins (x-axis), each containing an equal number of genes. The central dot represents the median, with gray box edges marking the 25th and 75th percentiles. Genes in the top bins were classified as zygotic (NTR > 70%, 75%, 80%, 85%), while those in the bottom bins were classified as maternal (NTR < 5%). The lower NTR cutoff is indicated by a red dashed line, and upper NTR cutoffs are shown with blue dashed lines. Genes with fewer than 10 UMI counts were filtered out across all five datasets.

b. Stacked bar chart showing proportions of identified maternal, maternal-zygotic, and zygotic genes (NTR > 75%, 80%, 85%) across three chemical conversion methods in our study compared to published data[7,8]. Different bar colors represent distinct gene types.

c. Venn diagram showing the overlap of defined zygotic genes (from b) among different chemical conversion methods and published studies[7,8], highlighting both unique and shared genes. Colors represent the data sources from various chemical conversion methods or published studies.

d. Box plot showing nucleotide substitution rates in our data compared to published data[7,8]. The colored boxes represent different platforms and treatment methods, with box edges corresponding to the 25th and 75th percentiles. The x-axis shows different types of base conversions, while the y-axis represents conversion rates. Source data are provided as a Source Data file.

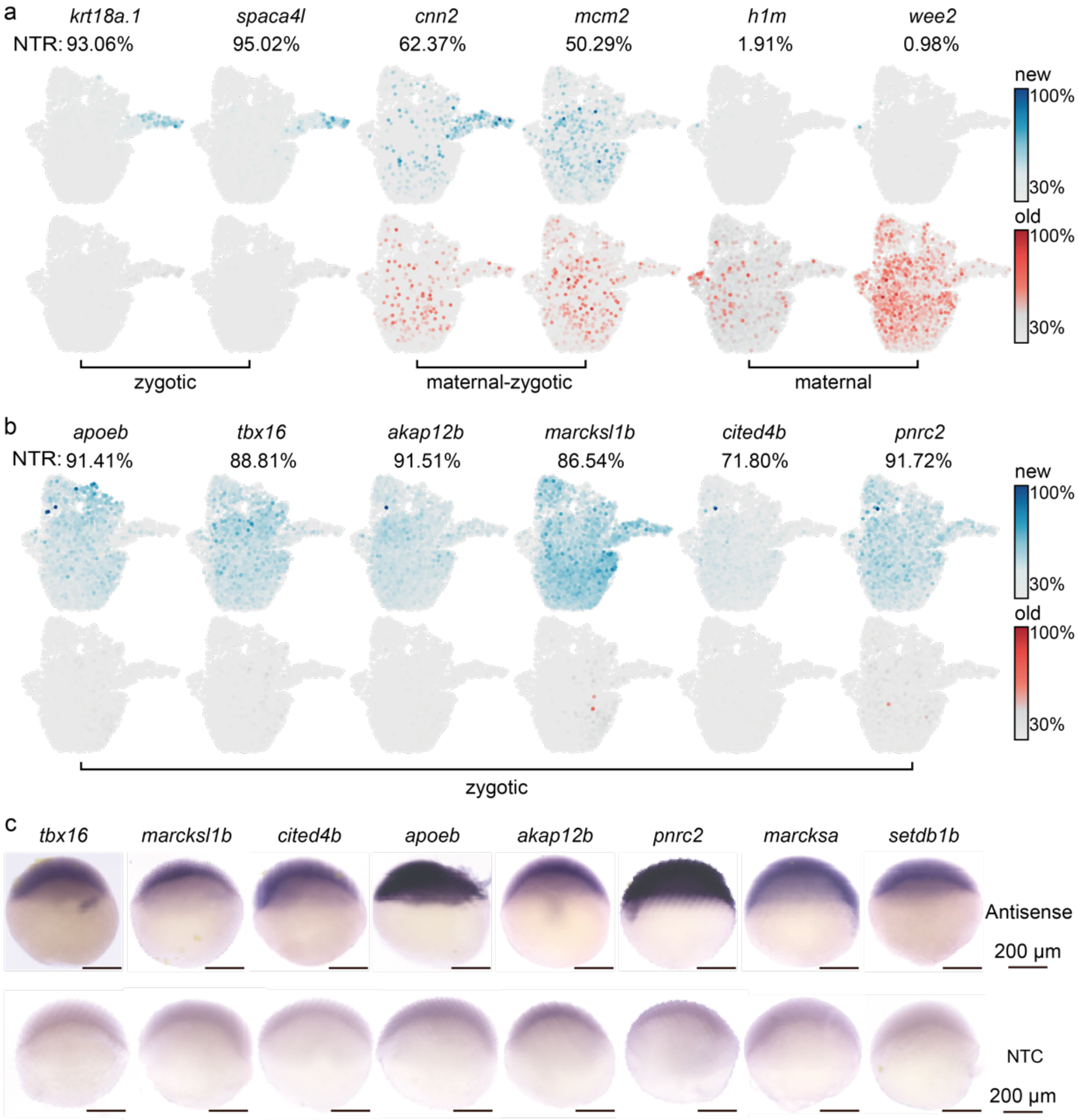
a
krt18a.1
spaca4l
cnn2
mcm2
h1m
wee2
NTR: 93.06%
95.02%
62.37%
50.29%
1.91%
0.98%
new
100%
30%
old
100%
30%
zygotic
maternal-zygotic
maternal
b
apoeb
tbx16
akap12b
marcksl1b
cited4b
pnrc2
NTR: 91.41%
88.81%
91.51%
86.54%
71.80%
91.72%
new
100%
30%
old
100%
30%
zygotic
c
tbx16
marcksl1b
cited4b
apoeb
akap12b
pnrc2
marcksa
setdb1b
Antisense
200 μm
NTC
200 μm

Supplementary Fig. 8 | Cell type transitions of 5.5 hpf zebrafish embryo.

a-b. Uniform Manifold Approximation and Projection (UMAP) projection depicting the proportions of zygotic (new) and maternal (old) transcripts for representative zygotic, maternal-zygotic and maternal genes (a), alongside the overlap of defined zygotic genes among different chemical conversion methods and published studies[7,8] (b), corresponding to Fig. 3f. Cells are color-coded by the normalized expression of newly transcribed zygotic copies (blue, top map) or pre-existing maternal copies (red, bottom map). The gene names with corresponding NTR values are indicated at the top, and the classification of genes is provided at the bottom.

c. Antisense (top) and sense-strand negative control (NTC, bottom) whole-mount in situ hybridization staining of 5.5 hpf zebrafish embryos, validating the expression of zygotic mRNAs shown in Fig. 3f,g. Scale bar: 200 µm. *tbx16*, *marcksl1b*, and *cited4b* are consistently identified across all datasets. *apoeb* is uniquely detected using on-beads methods across four datasets but absent in the in-situ chemical conversion study by Holler et al.[8]. *akap12b* is identified in four datasets, except for the study by Fishman et al.[7]. *pnrc2* is exclusively detected with our mCPBA/TFEA method (pH 7.4), while *marcksa* and *setdb1b* are uniquely identified using mCPBA/TFEA (pH 5.2 and pH 7.4). Each staining pattern was visualized in three independent samples and yielded similar results.

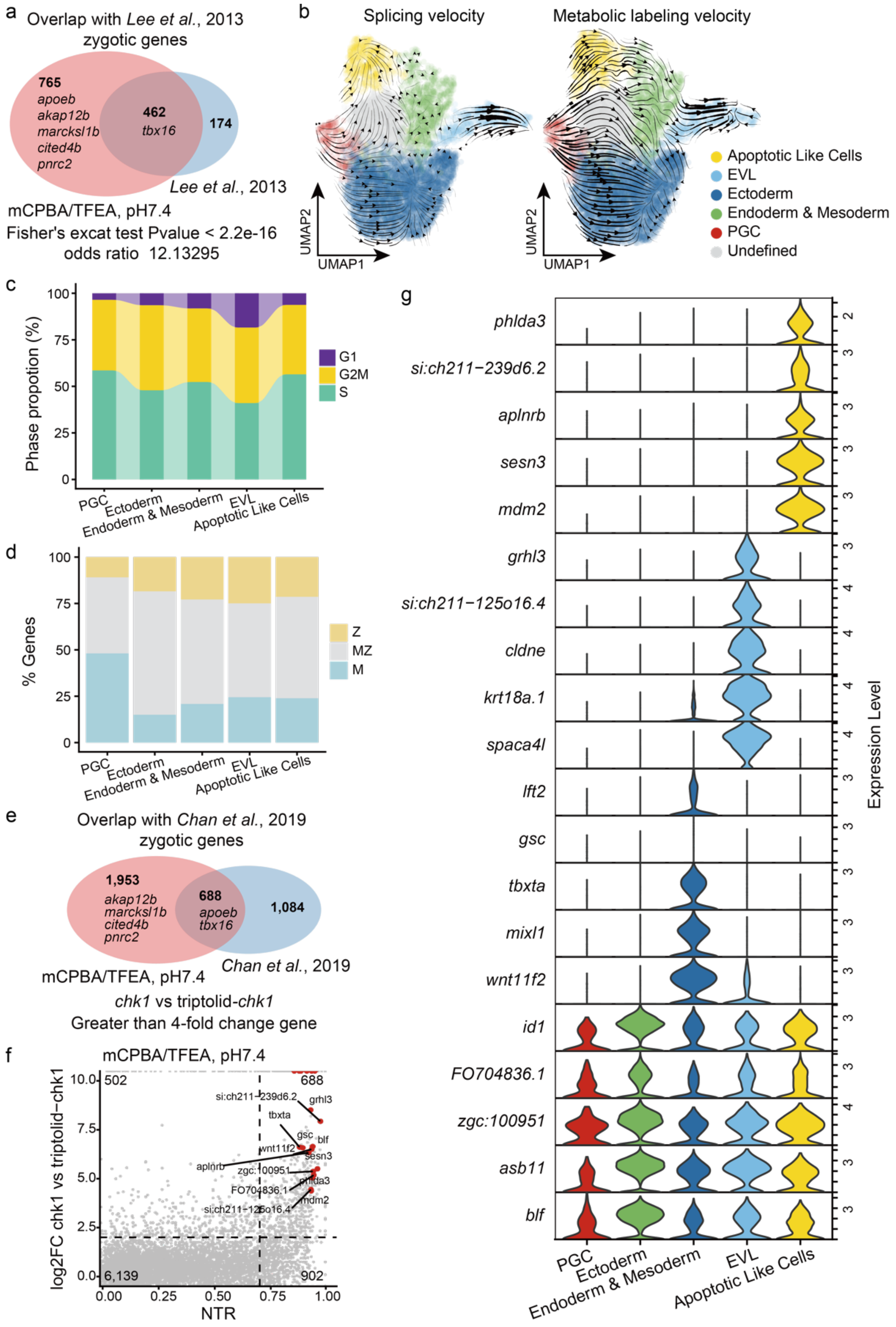
a
Overlap with Lee et al., 2013 zygotic genes
765
apoeb
akap12b
marcksl1b
cited4b
pnrc2
462
tbx16
174
Lee et al., 2013
mCPBA/TFEA, pH7.4
Fisher's excat test Pvalue < 2.2e-16
odds ratio 12.13295
b
Splicing velocity
Metabolic labeling velocity
UMAP1
UMAP2
Apoptotic Like Cells
EVL
Ectoderm
Endoderm & Mesoderm
PGC
Undefined
c
Phase propotion (%)
G1
G2M
S
PGC
Ectoderm
Endoderm & Mesoderm
EVL
Apoptotic Like Cells
d
% Genes
Z
MZ
M
e
Overlap with Chan et al., 2019 zygotic genes
1,953
akap12b
marcksl1b
cited4b
pnrc2
688
apoeb
tbx16
1,084
mCPBA/TFEA, pH7.4
Chan et al., 2019
chk1 vs triptolid-chk1
Greater than 4-fold change gene
f
mCPBA/TFEA, pH7.4
log2FC chk1 vs triptolid−chk1
NTR
502
688
6,139
902
g
phlda3
si:ch211−239d6.2
aplnrb
sesn3
mdm2
grhl3
si:ch211−125o16.4
cldne
krt18a.1
spaca4l
lft2
gsc
tbxta
mixl1
wnt11f2
id1
FO704836.1
zgc:100951
asb11
blf
Expression Level

Supplementary Fig. 9 | The expression of genes related to cell cycle length in differernt cell types of zebrafish embryos.

a. Venn diagram showing the overlap of zygotic genes identified using the mCPBA-based method (pH 7.4) and published studies[40], highlighting both unique and shared genes.

b. UMAP visualization of cell-type transitions using RNA velocity analysis in 5.5 hpf zebrafish embryos, integrating three chemical conversion methods, including on-beads IAA and mCPBA/TFEA (pH 5.2 and pH 7.4). The left panel shows velocity analysis using spliced and unspliced RNA matrices, while the right panel presents velocity analysis using new and total RNA matrices. Cells are color-coded by cell type, with streamlines indicating integration paths that connect local projections from the observed state to the extrapolated future state, and the thickness representing velocity magnitude. Arrows indicate the direction of subtype transitions computed by dynamo. EVL: enveloping layer; PGC: primordial germ cell.

c. Stacked bar chart showing the distribution of cell cycle phases (G1, S, and G2/M) across different cell types. Colors indicate distinct cell cycle phases.

d. Stacked bar chart showing the proportions of identified maternal (M), maternal-zygotic (MZ), and zygotic (Z) genes across different cell types. Colors represent different gene classifications.

e. Venn diagram showing the overlap of zygotic genes identified using the mCPBA-based method (pH 7.4) and genes upregulated by more than 4-fold in Chk1 overexpression embryos from a zebrafish cell cycle arrest model study[42], highlighting both unique and shared genes.

f. Scatter plot showing gene classification by plotting new-to-total RNA ratio (NTR) on the x-axis against gene induction in Chk1-overexpressing embryos on the y-axis. The horizontal dashed line marks a 4-fold change cutoff (Chk1 OE vs. triptolide-treated Chk1 OE), and the vertical line indicates a 70% NTR threshold. In the plot, 688 genes with >70% NTR are identified as zygotic and also exhibit up-regulation in Chk1 OE embryos with a prolonged cell cycle. Red dots highlight representative genes within this set.

g. Violin plots showing the induction of zygotic genes by cell cycle arrest across different cell types in zebrafish embryos. Colors represent distinct cell types.

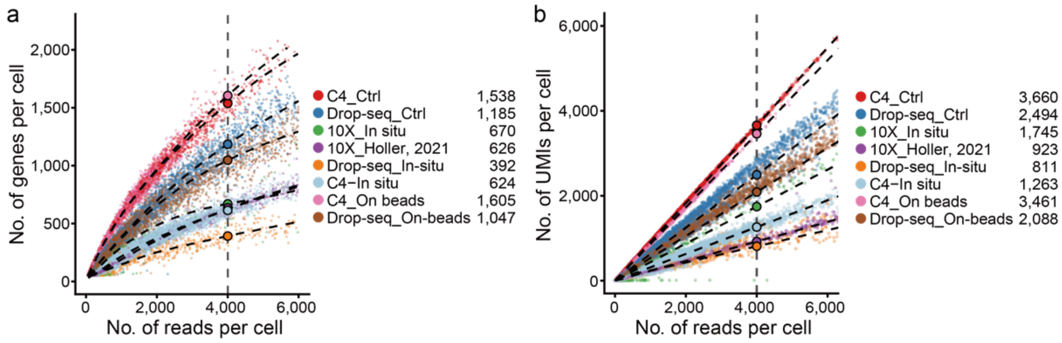

Supplementary Fig. 10 | Comparison between 10x Genomics, Drop-seq and MGI C4 high-throughput single-cell platforms.

a-b. Scatterplots showing the number of genes (a) or UMIs (b) detected per cell as a function of aligned reads per cell across the different platforms. Different colored dots represent various platforms and treatment methods. Fitted lines and predicted numbers of genes or UMIs detected per cell at 4,000 reads are shown for each platform. The predicted values for 4,000 reads are displayed in the upper right of the figure. The curve in a is smoothed using locally weighted regression, while in b is smoothed using a linear model. "Ctrl" represents the control group without chemical treatment; "In-situ" refers to “In-situ IAA, pH8.0” method, while “on-beads” indicates “On-beads IAA, 32°C” chemistry. To evaluate library complexity using in-situ IAA chemistry with the 10x Genomics platform, we retrieved raw data from Holler et al., 2021[8]. This study applied in-situ IAA chemistry to zebrafish embryos and performed library preparation using the 10x Genomics platform. The raw data was then processed using the same computational pipeline to ensure consistency in the analysis.

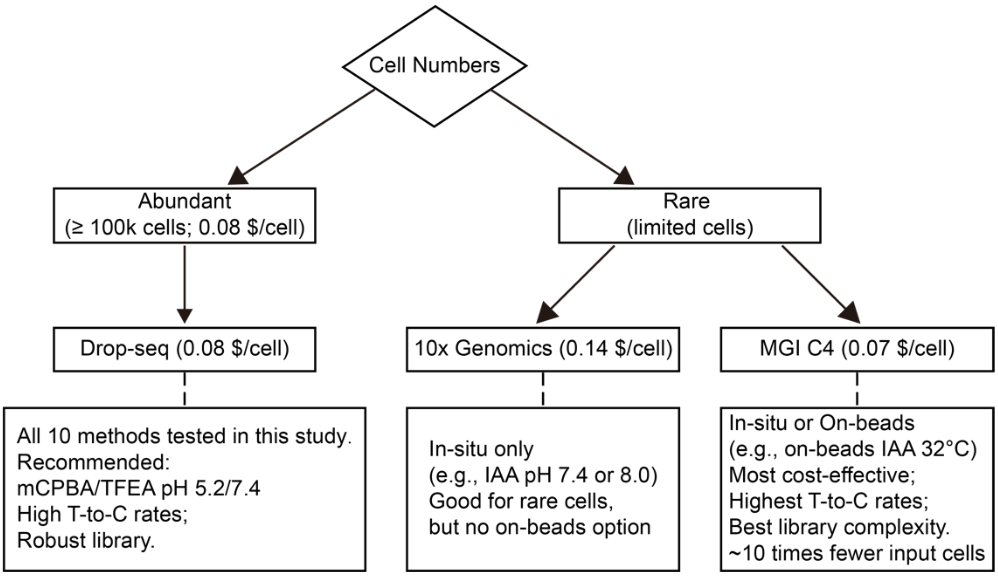

Supplementary Fig. 11 | Decision tree summary of chemical conversion methods and platform selection.

The cost estimates are based on prices in mainland China, including additional expenses such as taxes, and may be subject to inflation.

Supplementary Table 1: Library comparison of different samples in this study.

scRNA-seq platform	Sample name	Sample type	Labeling condition	T-to-C Substitution Rate (%)	Library Complexity (nGene @ 10k Reads/Cell)	Library Complexity (nUMI @ 10k Reads/Cell)	Library Complexity (nGene @ 4k Reads/Cell)	Library Complexity (nUMI @ 4k Reads/Cell)	Related data	Library preparation	Equipments	Library cost in China (US$/cell)
Drop-seq	Ctrl	ZF4	100 µM 4sU, 4 h	0.19	2,353	6,260	NA	NA	Fig. 2; Supplementary Fig. 2-6	2nd SS scNT-Seq	Drop-Seq microfludics platform	~0.08 (0.6 CNY/cell)
	In-situ IAA, pH 7.4	ZF4	100 µM 4sU, 4 h	1.71	913	4,723						
	In-situ IAA, pH 8.0	ZF4	100 µM 4sU, 4 h	3.53	740	1,736						
	On-beads IAA, 32°C	ZF4	100 µM 4sU, 4 h	6.57	2,137	5,362						
	On-beads IAA, 37°C	ZF4	100 µM 4sU, 4 h	3.84	2,129	4,116						
	mCBPA/TFEA, pH 5.2	ZF4	100 µM 4sU, 4 h	8.11	2,044	5,468						
	mCPBA/TFEA, pH 7.4	ZF4	100 µM 4sU, 4 h	8.40	1,747	4,350						
	NaIO4/TFEA, pH 5.2	ZF4	100 µM 4sU, 4 h	8.19	2,033	4,365						
	NaIO4/TFEA, pH 7.4	ZF4	100 µM 4sU, 4 h	6.75	1,838	5,310						
	NaIO4/NH4Cl, pH 8.8	ZF4	100 µM 4sU, 4 h	2.33	2,046	4,973						
	OsO4/NH4Cl, pH 8.8	ZF4	100 µM 4sU, 4 h	4.35	2,238	5,052						
10x Genomics	10x_In-situ	ZF4	100 µM 4sU, 4 h	1.84	NA	NA	670	1,745	Fig. 4; Supplementary Fig. 10	Chromium Next GEM Single Cell 3' Reagent Kits v3.1	10x Genomics Chromium Controller	~0.14 (1 CNY/cell)
MGI C4	C4_Ctrl	ZF4	100 µM 4sU, 4 h	0.29			1,538	3,660	Fig. 4; Supplementary Fig. 10	DNBelab C Series Single-Cell Library Prep set	C4 equipments	~0.07 (0.5 CNY/cell)
	C4_In situ	ZF4	100 µM 4sU, 4 h	5.74			624	1,263				
	C4_On-beads	ZF4	100 µM 4sU, 4 h	8.44			1,605	3,461				
Drop-seq	C4_Ctrl	ZF4	100 µM 4sU, 4 h	0.19			1,185	2,494	Fig. 4; Supplementary Fig. 10	2nd SS scNT-Seq	Drop-Seq microfludics platform	~0.08 (0.6 CNY/cell)
	C4_In situ	ZF4	100 µM 4sU, 4 h	3.53			392	811				
	C4_On-beads	ZF4	100 µM 4sU, 4 h	6.57			1,047	2,088				

Note: #nGene/cell: average number of genes detected per cell; #nUMI/cell: average number of UMIs detected per cell; #Reads/cell: average number of aligned reads per cell.

Supplementary Table 2: Labeled UMIs proportion of cycling and housekeeping genes.

Gene	Methods	Labeled UMIs proportion in one cell (%)	Labeld UMIs/Total UMIs	Related data
tubb4b (cycling)	Ctrl	1.79	1/56	Fig. 2
	In-situ IAA, pH 7.4	3.70	2/54	
	In-situ IAA, pH 8.0	20.00	3/15	
	On-beads IAA, 32°C	16.33	8/49	
	On-beads IAA, 37°C	15.69	8/51	
	mCBPA/TFEA, pH 5.2	31.82	14/44	
	mCPBA/TFEA, pH 7.4	36.76	25/68	
	NaIO4/TFEA, pH 5.2	32.08	17/53	
	NaIO4/TFEA, pH 7.4	22.03	13/59	
	NaIO4/NH4Cl, pH 8.8	10.00	5/50	
	OsO4/NH4Cl, pH 8.8	24.32	9/37	
ccnd1 (cycling)	Ctrl	3.12	1/32	Supplementary Fig. 5
	In-situ IAA, pH 7.4	22.22	2/9	
	In-situ IAA, pH 8.0	77.78	7/9	
	On-beads IAA, 32°C	100.00	17/17	
	On-beads IAA, 37°C	95.65	22/23	
	mCBPA/TFEA, pH 5.2	100.00	27/27	
	mCPBA/TFEA, pH 7.4	100.00	22/22	
	NaIO4/TFEA, pH 5.2	100.00	12/12	
	NaIO4/TFEA, pH 7.4	93.33	14/15	
	NaIO4/NH4Cl, pH 8.8	60.71	17/28	
	OsO4/NH4Cl, pH 8.8	93.75	15/16	
rplp0 (housekeeping)	Ctrl	0.00	0/65	Supplementary Fig. 5
	In-situ IAA, pH 7.4	0.00	0/54	
	In-situ IAA, pH 8.0	5.36	3/56	
	On-beads IAA, 32°C	1.54	1/65	
	On-beads IAA, 37°C	5.97	4/67	
	mCBPA/TFEA, pH 5.2	7.55	4/53	
	mCPBA/TFEA, pH 7.4	4.35	2/46	
	NaIO4/TFEA, pH 5.2	10.81	4/37	
	NaIO4/TFEA, pH 7.4	10.87	5/46	
	NaIO4/NH4Cl, pH 8.8	7.41	4/54	
	OsO4/NH4Cl, pH 8.8	2.82	2/71	

Supplementary Table 3: Comparison of chemical conversion methods and scRNA-seq platforms in this study.

Chemical Conversion Method	T-to-C Substitution Rate (%)	Library Complexity (nGene, nUMI @ 4k Reads/Cell)	Compatible Platforms	Capture Efficiency (%)	Cost per Cell ($)	Best Use Case
In-Situ IAA (pH7.4)	1.71	913 genes; 4,723 UMIs	10x Genomics, MGI C4, Drop-seq	High (~50%)	depends on the scRNA-seq platform	Widest compatibility and moderate sensitivity, best for commercial platforms
In-Situ IAA (pH8.0)	3.53	740 genes; 1,736 UMIs	10x Genomics, MGI C4, Drop-seq	High (~50%)		
On-Beads IAA (32°C)	6.57	2,137 genes; 5,362 UMIs	MGI C4, Drop-seq	High (~50%)		Optimal for high sensitivity, rare cell types, robust transcript recovery
On-beads mCPBA/TFEA (pH5.2)	8.11	2,044 genes; 5,468 UMIs	Drop-seq	Low (~5%)		Good balance between substitution rate and complexity, high-throughput
On-beads mCPBA/TFEA (pH7.4)	8.40	1,747 genes; 4,350 UMIs	Drop-seq	Low (~5%)		

scRNA-seq Platform	T-to-C Substitution Rate (%)	Library Complexity (nGene, nUMI @4k reads/cell)	Capture Efficiency	Chemical Conversion Compatibility	Cost per Cell ($)	Best Use Case
MGI C4	8.44% (Highest)	624 genes; 1,263 UMIs	~50% (High)	In-situ & On-beads IAA methods	0.07	Best for high sensitivity, rare cell populations, and optimal transcript recovery
10x Genomics	Lower than both MGI C4 & Drop-seq	670 genes; 1,745 UMIs	~50% (High)	In-situ only	0.14	Widely used, good alternative with moderate conversion efficiency
Drop-seq	6.57%	Lower than both MGI C4 & 10x	~5% (Low)	all methods in this study	0.08	Cost-effective, flexible, but lower capture efficiency

Supplementary Table 4: Primers sequences for whole-mount in situ hybridization.

Gene name	Primer-Forward	Primer-Reverse	Corresponding Figure
apoeb-total	AGATGACCCCATACGCCTCT	TAATACGACTCACTATAGGGGCGGATCTTCTGGGAGTA	Fig. 3g
tbx16-total	AACCTTTACCTTCCCCGAGA	TAATACGACTCACTATAGGGACGTTTCCATGGTGATAGCC	
akap12b-total	CAAGCAGTAGAGCCTGATGG	TAATACGACTCACTATAGGGTTACAAGAGGGGACTCAGCA	
marcksl1b-total	CGTCAAGACTAACGGACAGG	TAATACGACTCACTATAGGGGCAGGTGTTGAGTTGGTC	
cited4b-total	ATGATGATGCCCATGAACCA	TAATACGACTCACTATAGGGGTCCAGTCCTAGCTCCATGA	
pnrc2-total	CAACATTCCAGATCGCCCAG	TAATACGACTCACTATAGGGGGCTCACTAAACTTGGCTCC	
tbx16-intron	CCATCAAATCCCACTTCCACCCCA	TAATACGACTCACTATAGGGACATGAAATTGTCGGAAAAG	Fig. S8c
marcksl1b-intron	GTGTGGCGAGCAGACGGTGAT	TAATACGACTCACTATAGGGAGCGAAGCACGATGGAGAAT	
cited4b-intron	CGCTTGCGATGTCTGAATGCTGTC	TAATACGACTCACTATAGGGAAGGTGCAGAGTAGTCAGTAAC	
apoeb-intron	GGATGCTCCTTCAGGTGGCTTACG	TAATACGACTCACTATAGGGTGCGTAGGTTCTCGGCTG	
akap12b-intron	GCAGTCTCTTGCTAGGTTGGATGTCA	TAATACGACTCACTATAGGGATCGTCTGTGTGTTTGGTCTC	
pnrc2-intron	AACAACGCTCACTGGCACATGAA	TAATACGACTCACTATAGGGCTAACCGGCTGGCTCTTC	
marcksa-intron	CAGAGACCCAAATCAACTACCACACAC	TAATACGACTCACTATAGGGTTGTCAGGAATGCCAGTG	
setdb1b-intron	GAAGTGGGTCATAGCAGGCAGAATT	TAATACGACTCACTATAGGGTGGAGCAAGCAAGCACATC	
NTC-tbx16-intron	TAATACGACTCACTATAGGGCCATCAAATCCCACTTCCACCCCA	ACATGAAATTGTCGGAAAAG	
NTC-marcksl1b-intron	TAATACGACTCACTATAGGGGTGTGGCGAGCAGACGGTGAT	AGCGAAGCACGATGGAGAAT	
NTC-cited4b-intron	TAATACGACTCACTATAGGGCGCTTGCGATGTCTGAATGCTGTC	AAGGTGCAGAGTAGTCAGTAAC	
NTC-apoeb-intron	TAATACGACTCACTATAGGGGGATGCTCCTTCAGGTGGCTTACG	TGCGTAGGTTCTCGGCTG	
NTC-akap12b-intron	TAATACGACTCACTATAGGGGCAGTCTCTTGCTAGGTTGGATGTCA	ATCGTCTGTGTGTTTGGTCTC	
NTC-pnrc2-intron	TAATACGACTCACTATAGGGAACAACGCTCACTGGCACATGAA	CTAACCGGCTGGCTCTTC	
NTC-marcksa-intron	TAATACGACTCACTATAGGGCAGAGACCCAAATCAACTACCACACAC	TTGTCAGGAATGCCAGTG	
NTC-setdb1b-intron	TAATACGACTCACTATAGGGGAAGTGGGTCATAGCAGGCAGAATT	TGGAGCAAGCAAGCACATC	
