# Supplementary material for: Benchmarking metabolic RNA labeling techniques for high-throughput single-cell RNA sequencing
Source: Nat Commun. 2025 Jul 1;16:5952. doi: 10.1038/s41467-025-61375-z (PMC12215390; doi:10.1038/s41467-025-61375-z)
Supplement: Supplementary file 2 — Reporting Summary [file 41467_2025_61375_MOESM2_ESM.pdf]

nature portfolio

Corresponding author(s): Peng Hu

Last updated by author(s): May 28, 2025

Reporting Summary

Nature Portfolio wishes to improve the reproducibility of the work that we publish. This form provides structure for consistency and transparency in reporting. For further information on Nature Portfolio policies, see our Editorial Policies and the Editorial Policy Checklist.

Statistics

For all statistical analyses, confirm that the following items are present in the figure legend, table legend, main text, or Methods section.

n/a	Confirmed	
☐	☒	The exact sample size (n) for each experimental group/condition, given as a discrete number and unit of measurement
☐	☒	A statement on whether measurements were taken from distinct samples or whether the same sample was measured repeatedly
☐	☒	The statistical test(s) used AND whether they are one- or two-sided<br>*Only common tests should be described solely by name; describe more complex techniques in the Methods section.*
☒	☐	A description of all covariates tested
☐	☒	A description of any assumptions or corrections, such as tests of normality and adjustment for multiple comparisons
☐	☒	A full description of the statistical parameters including central tendency (e.g. means) or other basic estimates (e.g. regression coefficient) AND variation (e.g. standard deviation) or associated estimates of uncertainty (e.g. confidence intervals)
☐	☒	For null hypothesis testing, the test statistic (e.g. F, t, r) with confidence intervals, effect sizes, degrees of freedom and P value noted<br>*Give P values as exact values whenever suitable.*
☒	☐	For Bayesian analysis, information on the choice of priors and Markov chain Monte Carlo settings
☒	☐	For hierarchical and complex designs, identification of the appropriate level for tests and full reporting of outcomes
☐	☒	Estimates of effect sizes (e.g. Cohen's d, Pearson's r), indicating how they were calculated

Our web collection on statistics for biologists contains articles on many of the points above.

Software and code

Policy information about availability of computer code

Data collection	The data used in this study were generated using sequencer (Illumina) or collected from experiments.
Data analysis	dynast (version 1.0.1), fastp (version 0.20.1), cutadapt (version 4.2), hista2 (version 2.2.1), featureCounts (version 2.0.3), DNBC4tools (version 2.1.1), R (version 4.2.0), R packages: dplyr (version 1.1.3), tidyr (version 1.3.0), reshape (version 0.8.9), RColorBrewer (version 1.1.3), clusterProfiler (version 4.6.2), ggvennDiagra (version 1.5.2), ggalluvial (version 0.12.5), cowplot (version 1.1.1), ComplexHeatmap (version 2.14.0), Python (version 3.8.18), Python packages:matplotlib (version 3.6.3), Scanpy (version 1.9.6), dynamo (version 1.4.0), omicverse (version 1.5.7), numpy (version 1.23.5), pandas (version 1.5.3), anndata (version 0.9.2). All scripts for figure generation are available at https://github.com/penghu-sc/Benchmarking-Metabolic-RNA-Labeling.

For manuscripts utilizing custom algorithms or software that are central to the research but not yet described in published literature, software must be made available to editors and reviewers. We strongly encourage code deposition in a community repository (e.g. GitHub). See the Nature Portfolio guidelines for submitting code & software for further information.

Data

Policy information about availability of data

All manuscripts must include a data availability statement. This statement should provide the following information, where applicable:

- Accession codes, unique identifiers, or web links for publicly available datasets
- A description of any restrictions on data availability
- For clinical datasets or third party data, please ensure that the statement adheres to our policy

Raw data have been deposited in National Genomics Data Center database under accession No. PRINA1149934 (https://ngdc.cncb.ac.cn/bioproject/browse/PRJCA037071).

Research involving human participants, their data, or biological material

Policy information about studies with human participants or human data. See also policy information about sex, gender (identity/presentation), and sexual orientation and race, ethnicity and racism.

Reporting on sex and gender	No human samples were used in this study.
Reporting on race, ethnicity, or other socially relevant groupings	No human samples were used in this study.
Population characteristics	No human samples were used in this study.
Recruitment	No human samples were used in this study.
Ethics oversight	No human samples were used in this study.

Note that full information on the approval of the study protocol must also be provided in the manuscript.

Field-specific reporting

Please select the one below that is the best fit for your research. If you are not sure, read the appropriate sections before making your selection.

☒ Life sciences ☐ Behavioural & social sciences ☐ Ecological, evolutionary & environmental sciences

For a reference copy of the document with all sections, see nature.com/documents/nr-reporting-summary-flat.pdf

Life sciences study design

All studies must disclose on these points even when the disclosure is negative.

Sample size	Cell numbers commonly used in the field were adopted, as reported in prior studies (Hu et al., 2017, Molecular Cell; Fishman et al., 2024, Nature Communications). More than 1,500 cells were profiled per experiment to benchmark the T-to-C conversion efficiency across seven chemical conversion methods. Additionally, more than 1,500 cells were analyzed in each of the four experiments conducted on zebrafish embryos at 5.5 hours post-fertilization (hpf).
Data exclusions	Data were not excluded.
Replication	All experiments were performed across hundreds to tens of thousands of individual cells for ZF4 cell line datasets. For Drop-seq, the number of cells captured for each condition was as follows: control (7,531 cells), in situ IAA at pH 7.4 (1,587 cells), in-situ IAA at pH 8.0 (789 cells), on-beads IAA at 32°C (5,581 cells), on-beads IAA at 37°C (5,267 cells), mCPBA/TEEA at pH 5.2 (4,692 cells), mCPBA/TFEA at pH 7.4 (4,639 cells), NaIO4/TEEA at pH5.2 (5,360 cells), Nal04/TEEA at pH 7.4 (6,461 cells), Nal04/NH4Cl at pH 8.8 (5,389 cells) and 0s04/NH4Cl at pH 8.8 (5,233 cells). For MGI C4, t he number of cells captured for each condition was as follows: control (4,934 cells), in-situ IAA at pH 8.0 (18,132 cells) and on-beads IAA at 32°C (4,921 cells). For 10x Genomics, the in situ IAA at pH 8.0 methods captured 7,578 cells.<br>No biologically replication was performed for the zebrafish embryos datasets. For Drop-seq, approximately 200 embryos were used per group, and cell counts for each condition were: control (1,997 cells), on-beads IAA (1,699 cells), mCPBA/TFEA at pH 7.4 (3,456 cells) and mCPBA/TFEA at pH 5.2 (2,731 cells).
Randomization	No randomization strategies were applied.
Blinding	Not applied. Blinding was not relevant since sample identities were encoded into experiment design.

Reporting for specific materials, systems and methods

We require information from authors about some types of materials, experimental systems and methods used in many studies. Here, indicate whether each material, system or method listed is relevant to your study. If you are not sure if a list item applies to your research, read the appropriate section before selecting a response.

Materials & experimental systems

n/a	Involved in the study	
☐	☒	Antibodies
☐	☒	Eukaryotic cell lines
☒	☐	Palaeontology and archaeology
☐	☒	Animals and other organisms
☒	☐	Clinical data
☒	☐	Dual use research of concern
☒	☐	Plants

Methods

n/a	Involved in the study	
☒	☐	ChIP-seq
☐	☒	Flow cytometry
☒	☐	MRI-based neuroimaging

Antibodies

Antibodies used	The primary antibody used in whole-mount in situ hybridization included anti-digoxigenin (DIG) antibody (Roche, 11093274910).
Validation	The antibody used in this study are from commercial suppliers that have verified the specificity of the antibody, which has been previously used by various laboratories.

Eukaryotic cell lines

Policy information about cell lines and Sex and Gender in Research

Cell line source(s)	ZF4 (CZRC, Cell1) were originally purchased from China Zebrafish Resource Center. The ZF4 cell line was established from 1-day-old zebrafish embryos.
Authentication	ZF4 (CZRC, Cell1) were originally purchased from China Zebrafish Resource Center and no additional authentication was performed.
Mycoplasma contamination	Not tested.
Commonly misidentified lines (See ICLAC register)	No commonly misidentified cell lines were used.

Animals and other research organisms

Policy information about studies involving animals; ARRIVE guidelines recommended for reporting animal research, and Sex and Gender in Research

Laboratory animals	Wildtype zebrafish AB and TU strains used in this study were purchased from the China Zebrafish Resource Center, National Aquatic Biological Resource Center, CZRC/NABRC.
Wild animals	This study did not involve wild animals.
Reporting on sex	Sex was not considered in this study, as the embryos sex are not determined at the early developmental stages examined in this work (5.5 hpf).
Field-collected samples	This study did not involve field-collected samples.
Ethics oversight	All fish were maintained and experiments conducted in accordance with protocols reviewed and approved by the Animal Ethics Committee on Laboratory Animal Care and Use of Shanghai Ocean University, with approval granted on February 26, 2022.

Note that full information on the approval of the study protocol must also be provided in the manuscript.

Plants

Seed stocks	No plant samples were used in this study.
Novel plant genotypes	No plant samples were used in this study.
Authentication	No plant samples were used in this study.

Flow Cytometry

Plots

Confirm that:

- [x] The axis labels state the marker and fluorochrome used (e.g. CD4-FITC).
- [x] The axis scales are clearly visible. Include numbers along axes only for bottom left plot of group (a 'group' is an analysis of identical markers).
- [ ] All plots are contour plots with outliers or pseudocolor plots.
- [x] A numerical value for number of cells or percentage (with statistics) is provided.

Methodology

Sample preparation	After 4sU labeling, ZF4 cells were dissociated into single cell suspension. The cells were counted, and aliquots of 1 million cells were dispensed into labeled tubes, followed by washing with DPBS and centrifugation for 5 minutes at 800 rpm. For fixation, the cell pellet was resuspended in 300 µL PBS with gentle vortex, followed by the addition of 700 µL ice cold ethanol dropwise while continuously vortexing. The mixture was then incubated at 4 °C for 30 minutes to overnight. After fixation, the cells were centrifuged and resuspended in 250 µL of DPBS followed by the addition 5 µL of 10mg/mL RNase A (Sigma-Aldrich, R-6513) to a final concentration of 0.2-0.5 mg/mL. The cells were then Incubated at 37 °C for one hour. Cells were then stained by adding 10 µL of a 1mg/mL propidium iodide (PI) solution (Sigma-Aldrich, P-4170) to a final concentration of 10 µg/mL, and kept in the dark for at least one hour until analysis. The cell cycle was analysed using the BD FACSMelody (BD Biosciences). Fractions of cells in each phase were quantified using FlowJo software V10.8.1.
Instrument	Flow cytometry was performed on BD FACSMelody (BD Biosciences).
Software	FlowJo V10.8.1
Cell population abundance	At least 10,000 cells were analyzed per group.
Gating strategy	During quantification, lymphocytes were identified by gating on forward-scatter area (FSC-A) and side-scatter area (SSC-A). Doublets were excluded using FSC-A versus forward-scatter height (FSC-H). Cohesive Cells were further exclude using PE-A versus PE-H (adjust the PE coordinates to linear). Using the Cell cycle plug-in to conduct cell cycle analysis: select the appropriate channel (PE-A) for the horizontal coordinate and select the appropriate fitting algorithm Watson (Pragmatic) in the Model.

- [x] Tick this box to confirm that a figure exemplifying the gating strategy is provided in the Supplementary Information.
